# Feasibility, acceptance, user satisfaction, and differences between users and non-users of an online-based support service for University students

**DOI:** 10.1007/s44192-026-00648-5

**Published:** 2026-11-11

**Authors:** Juliane Hug, Antonia Louise Schiel, Aneliana da Silva Prado, Elisabeth Kohls, Christine Rummel-Kluge

**Affiliations:** 1 Department of Psychiatry and Psychotherapy, Medical Faculty, Leipzig University, Leipzig, Germany; 2 Department of Psychiatry and Psychotherapy, University Leipzig Medical Center, Leipzig University, Leipzig, Germany

**Keywords:** University students, Mental health, Online counselling

## Abstract

Mental health problems among university students have increased markedly in recent years. Despite their prevalence, help-seeking remains low, highlighting the need for accessible, low-threshold support. Digital interventions, including online counselling and app-based tools, may help bridge this treatment gap. This study aimed to evaluate the feasibility and acceptability of the digital mental health platform “enhance” as a low-threshold support service for university students, with a particular focus on characterizing counselling users and examining differences between users and non-users. A pragmatic, self-selected survey-based cohort study was conducted across six German universities. Students voluntarily registered and completed standardized assessments at baseline (T0) and post-intervention (T1). The platform included a mental health app and individual counselling via chat, video, or email. Overall, 447 students completed baseline assessments (mean age = 24.15 years; 76.5% female). Moderate anxiety and depression levels were reported. Of these, 343 downloaded the app, 117 engaged in at least one counselling session, with 41% choosing video counselling for their first contact. Satisfaction was high (ZUF-8: M = 20.14); 95.9% reported improved well-being after sessions, and 78.4% found the intervention helpful. Most sessions addressed mental health concerns and resulted in referral recommendations (80.9​%). Higher eating disorder symptoms and having a parent with a migration history were associated with lower counselling uptake. Findings suggest that “enhance” is a feasible and well-accepted low-threshold intervention, however, only 26% of the baseline sample engaged with at least one counselling session, warranting further research on long-term effectiveness and integration into university support systems.

**Supplementary Information:**

The online version contains supplementary material available at https://doi.org/10.1007/s44192-026-00648-5.

## Study registration

DRKS00029467, Registration Date: 15.09.2022

**Supplementary Information:**

The online version contains supplementary material available at https://doi.org/10.1007/s44192-026-00648-5.

## Introduction

Mental health issues represent a growing concern in public health, with mental disorders being major contributors to disability worldwide [1]. The prevalence of mental health issues significantly increased during and in the aftermath of the COVID-19 pandemic, particularly among children, adolescents, and young adults [2].

In Germany, the proportion of university students reporting clinically relevant depressive symptoms increased from 29.0% in 2019 to 38.9% in 2020 and 40.7% in 2021. During the same period, generalized anxiety increased from 29.7% to 35.9%, while suicidal ideation increased from 16.0% to 21.4%, and levels of loneliness remained substantially elevated [3].

Research has since been increasingly undertaken on university students, highlighting them as a heavily affected group, e.g., showing a persistent rise in suicidal ideation, a reduction in reported social support and associated perceived loneliness during the years of the COVID-19 pandemic and beyond [4]. Even though mental disorders are very common amongst university students [5], only a small percentage of those affected seek professional help [6].

Mental health difficulties can lead to several negative outcomes, including poor academic performance, sub-optimal mental health, reduced study satisfaction, and dropout from study [5, 7]. Despite the high prevalence of mental health challenges found in the student population [8], students often do not seek professional help or are unaware of the psychosocial support services offered by their universities: A survey of over 5000 university students in Germany found that 17–19% were not willing to seek professional mental health help. In addition, 34–38% were unaware of their university’s psychosocial counselling services [9]. These circumstances underscore the need for easily accessible, low-threshold psychosocial care services, with internet- based interventions offering a promising option by facilitation early support and engaging individuals who may not utilize traditional mental health services [10].

Most web-based systems focus on the general population, and only a few address the everyday life context of higher-education students [11]. The existing online mental health programmes for students have shown promising feasibility, acceptability, and potential effectiveness across diverse formats [12]. For example, the CALM computerised CBT self-help programme has been positively received by higher-education students, who reported improvements in both personal well-being and academic life [13]. Additionally, a fully digital mental health literacy course demonstrated high acceptability, with participants showing significant improvement in mental health knowledge, emotional self-awareness, and reductions in substance use over 12 weeks [14]. Furthermore, a randomized controlled pilot study found that the COPE online cognitive-behavioral skill-building program was feasible and well-accepted among first-year college students; while overall anxiety and depressive symptoms did not differ significantly from the control group, students with elevated anxiety at baseline showed significant symptom reduction (State anxiety (STAI): mean decreased 18.70 points), and COPE participants also achieved higher grade point averages [15]. These findings are consistent with broader evidence supporting digital mental health interventions among university students. A systematic review of 89 digital mental health interventions for college students found that 47% demonstrated evidence of effectiveness, with interventions targeting depression, anxiety, and stress showing promising outcomes [16]. Importantly, however, only a small proportion of the studies examined implementation at the broader university-campus level, highlighting a gap between evidence of efficacy and evidence regarding real-world implementation. There is a growing consensus that accessible, low-threshold mental health interventions are essential within higher education institutions. Programs such as peer-support initiatives, campus mental health services, and digital interventions may therefore represent important strategies to address the mental health needs observed in recent years.

There is therefore growing evidence supporting digital interventions as accessible approaches to addressing mental health needs in higher education [16]. However, evidence on the feasibility, acceptability, and implementation of comprehensive, low-threshold digital mental health services within university settings remains comparatively limited. In particular, little is known about which students engage with such services and whether users differ from non-users in terms of sociodemographic characteristics, mental health symptoms, or previous help-seeking behavior [17].

Therefore, an online support program specifically tailored to address the unique needs of university students was developed. The online platform enhance (www.enhance-university.de) is a comprehensive digital support system for promoting university students’ mental health. Based on previous research conducted by the authors [4, 18] several online-based services were developed and implemented, including: A mental health app (m-path – developed by a research team at Katholieke University Leuven [19]), a chat-based individual psychological counseling, a video-based individual psychological counseling and an email-based individual psychological counselling. These services have been rolled out at six universities in the state of Saxony (Germany), starting October 2022 and was still ongoing by the time this manuscript was submitted.

The current study aimed to (a) characterize the users of a counselling service (chat, video, email) and (b) evaluate the feasibility and acceptability of the service, and (c) compare the difference between users and non-users on sociodemographic variables and mental health outcomes.

## Materials and methods

### Description of the online counselling service

Enhance was developed as an adjunct, primary- and secondary-preventive offering, available to students on demand – time slots could be booked online, service was available during working hours. These services were not meant to replace counselling or psychotherapy; rather, they were designed and implemented to complement existing care as a low-threshold entry point into the (mental) healthcare system or to appropriate referral services. The service offers four different and independent modalities which could be used: A mental health app (m-path); a chat-based individual psychological counseling; a video-based individual psychological counselling; and an e-mail-based individual psychological counselling. Users had the option to register for participation in one of the available modalities using enhance website user’s interface (https://enhance-university.de/). Following, each offer is described more in-depth.

#### App intervention

The app, built with m-path, is an integral part of the enhance program. M-path has been developed by a research team at Katholieke University Leuven with the goal of providing a flexible and customisable infrastructure for implementing smartphone-based ecological momentary assessment (EMA) and ecological momentary intervention (EMI) across research and clinical contexts within a blended-care framework [19]. The app was developed by the authors in collaboration with psychotherapists in training. It provides students with practical, evidence-informed modules, available in both written and audio formats, addressing key aspects of student well-being, including establishing routines, identifying personal resources, strengthening social connections, managing exam anxiety, improving time management, overcoming procrastination, and coping with performance-related stress. In addition, the app offers a range of relaxation and stress-management techniques, including guided breathing exercises, abdominal breathing, mindfulness-based breathing, progressive muscle relaxation, and guided imagery. Regional contact points provide information on local support services, while a dedicated emergency section offers guidance on accessing immediate assistance.The app was designed to reduce barriers to accessing existing university support services, including limited awareness of available resources and the time and effort required to identify an appropriate service. All intervention content and services available through the app were developed, compiled, and implemented by the project team. Access to the app content and participant usage data was restricted to authorised project staff. Thus, m-Path served solely as the technical infrastructure for the present project, enabling the development of an app with content tailored to the needs of university students.

The app could be used by all registered students on the enhance platform as they were provided with an indicidual app-code after registration.

#### Professional counselling via chat, video and email

The aim of the individual chat, video, and email counselling offered within enhance program was to provide psychoeducational guidance and, when applicable, to refer students to appropriate support services in and outside the university settings.

**Fig. 1:**
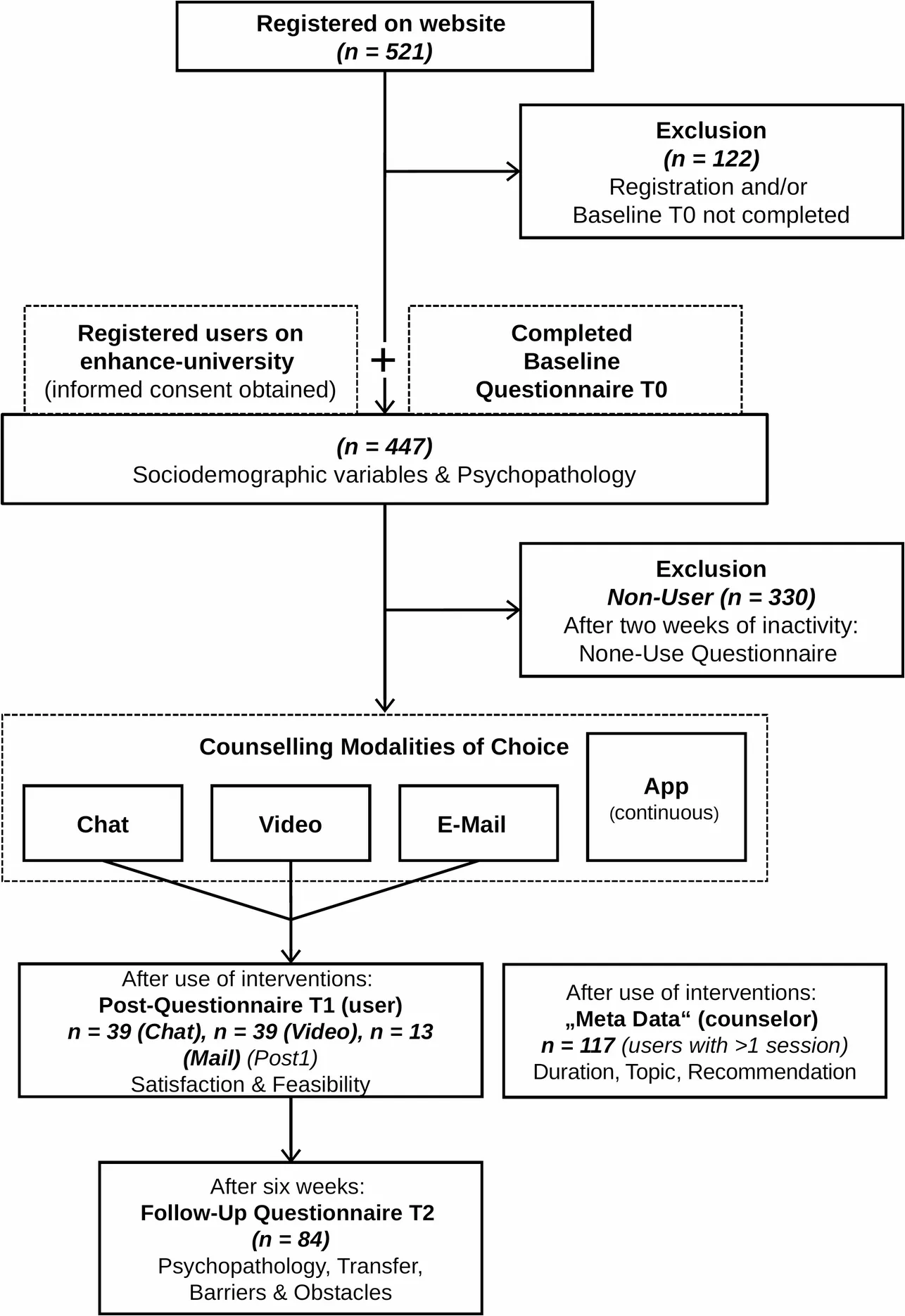
User flow and uptake of interventions in enhance

Chat-based services were delivered via the GDPR-compliant messaging service Signal (Fig. 1) (https://signal.org/#signal). Video consultations were conducted using the GDPR-compliant video consultation platform RED connect basis (https://redmedical.de/), certified by the National Association of Statutory Health Insurance Physicians (Germany). Email consultations were provided via a project email account of the Medical Faculty of Leipzig University.

The goal of the individual chat, video, and email counselling services was to provide psychoeducational guidance and to facilitate a targeted referral of the participants to suitable care or support services, if necessary. All services were conducted by psychologists (M.Sc. or diploma) and/or psychotherapists in training. The sessions were delivered by any of the four counsellors who received prior training.

In order to preserve the acute counselling character of the support services, participation restrictions had been defined. While video and chat counselling took place at pre-scheduled appointments and were limited to a duration of 30 min, email counselling continued until either the counsellor or the participant indicated that the concern had been resolved or a recommendation for further referral had been provided. In exceptional cases, these specifications could be exceeded if deemed necessary by the counsellor.

Participation in each modality was restricted to a maximum of three sessions over the course of the study.

### Study design

A pragmatic, cross-sectional self-selected cohort design was applied, in which students voluntarily enrolled in the online mental health program – enhance. All measures were assessed through standardized questionnaires at baseline (T0), post-intervention (administered immediately after the consultation; T1), and follow-up (administered after six weeks of completing baseline assessment; T2). For the purpose of this study, only outcomes at baseline (T0) and post-intervention (T1) time points were analyzed. Data was collected and analyzed between the launch of the programme on 1st November 2022 until 31st October 2025 (3 years in total).

### Participants and procedure

The study was conducted across six universities and higher-education institutions in Germany: Leipzig University (Universität Leipzig), Leipzig University of Music and Theatre (Hochschule für Musik und Theater Leipzig), Dresde University of Technology (Technische Universität Dresden), University of Applied Sciences for Social Work, Education and Nursing (Evangelische Hochschule Dresden), Hochschule Mittweida University of Applied Sciences; and Hochschule Meißen University of Applied Sciences (Hochschule Meißen, HSF Meißen). These institutions participated in the implementation and evaluation of the enhance intervention and had been subject of a student survey beforehand [4]. The participating institutions were selected from the population of all higher education institutions in the state of Saxony. To maximize the generalizability of the findings, institutions were selected using a structured process. All Saxon higher education institutions were categorized according to their organizational structure and subject profile into five groups: (1) universities, (2) universities of music and the arts, (3) universities of applied sciences, (4) specialized institutions, and (5) institutions offering dual study programs. From each category, the institutions with the largest student populations were identified for potential participation. Students enrolled at the participating institutions were eligible to use the intervention and were invited to participate in the accompanying evaluation study. Students ofthe respective universities were informed about the study and about the enhance program through campus postings, brochures, and information on student service websites, which included a link (www.enhance-university.de) and instructions to service registration.

Eligibility criteria included being a student aged 18 years or older, enrolled at one of the participating universities, providing informed consent, and possessing sufficient German language proficiency, as well as adequate visual and reading ability. In addition, participants were required to have internet access, a valid email address, and access to a smartphone or computer.

After receiving detailed study and data protection information, including downloadable study documents, participants provided electronic informed consent via an opt-in procedure. Subsequently, interested students registered using an email address and a self-selected password. Upon first login to the enhance platform, users were required to complete the baseline assessment (T0) to finalize registration and gain access to counselling services. Completion of the questionnaire generated a unique identifier, which enabled the linkage of survey data to the individual user profile within the platform. The ethics committee of the Leipzig University granted approval for the study on 15 August 2022 (file reference: 276/22-ek).

During the six-week period, users were administered questionnaires on satisfaction and perceived helpfulness directly following the use of a counselling intervention of either video, chat or mail (T1). After six weeks, participants were sent a follow-up questionnaire (T2) and reminded via email. For the purpose of this study, only baseline (T0) and post-intervention (T1) assessments were evaluated.

Participants who provided informed consent and completed registration and the baseline assessment (T0), but did not engage with any intervention within two weeks during the six-week study period, or who actively withdrew consent, were classified as dropouts. For an overview of uptake flow, see Fig. 1.

### Measures

#### Sociodemographic characteristics

Prior to commencing the intervention, participants were asked to provide basic sociodemographic information in the baseline questionnaire (T0), including age, gender, relationship, and employment status, living situation (alone or with other people), children living in the household, and psychological distress.

Concerning gender, the category “diverse” refers to participants who did not identify themselves exclusively as male or female and selected the “diverse” gender category in the baseline questionnaire. This category was intended to include participants with gender identities outside the binary categories of male and female.

#### Mental health measures

##### Depressive symptoms

Potential symptoms of depression were assessed at T0 using the Patient Health Questionaire-9 (PHQ-9). Participants were asked to report how frequently they have experienced depressive symptoms during the preceding two weeks. Each item is rated on a 4-point Likert scale ranging from 0 to 3 (0 = not at all; 1 = several days; 2 = more than half the days; 3 = nearly every day). The PHQ-9 total score was calculated by summing the nine item scores (range 0–27). Higher scores reflect greater severity of depressive symptomatology [20].

##### Anxiety symptoms

The participant’s anxiety symptoms were measured at T0 with the General Anxiety Disorder Questionaire (GAD-7). The seven-item questionnaire covers the frequency of anxiety symptoms during the past two weeks. Each item is rated on a 4-point Likert scale extending from 0 to 3 (0 = not at all; 1 = several days; 2 = more than half the days; 3 = nearly every day). The total score is obtained by summing the scores of the seven items and ranges from 0 to 21 [21].

##### Alcohol and substance consumption

Participants were asked about their alcohol and substance consumption at T0 using the Alcohol Use Disorders Identification Test (Audit-C). The Audit-C is a subset of the longer AUDIT, consisting of the first three items, which asses the frequency of alcohol consumption, the usual quantity of alcohol consumed per drinking day and the frequency of heavy drinking, i.e. consuming more than six drinks. A score between 0 and 4 is assigned to each question [22, 23]. The AUDIT-C score was calculated by summing the three item scores (range 0–12).

##### Eating disorder symptoms (EDE-Q8)

Dietary behaviour was evaluated at T0 with the Eating Disorder Examination Questionaire-8 (EDE-Q8). The form consists of eight items and uses a 7-point Likert scale ranging from 0 to 6 (0 = characteristic is not present; 6 = every day/ characteristic is present in an extreme form) referring to the past 28 days [24, 25]. For the EDE-Q8, each of the eight items is scored from 0 to 6. The EDE-Q8 score was calculated as the mean of the eight item scores, with higher scores indicating greater eating-disorder symptomatology.

All aforementioned measures have been previously used in large-scale online surveys with university students [9, 18, 26, 27].

#### Discrimination

Items evaluating experiences of discrimination were incorporated into the baseline questionnaire T0 and added to the present study in November 2024. Participants were asked whether they had experienced discrimination in the past 2 years for any of the following reasons: ethnicity or origin, gender, gender identity (e.g., transgender or intersex), disability or chronic illness, old age, being too young, sexual orientation (operationalised through sexual identity e.g., gay, lesbian, and bisexual), low level of education, or low income. For each category, participants could respond with “yes,” “no,” or “no answer.” An additional option allowed participants to indicate discrimination for another reason, which could be specified in a free-text field. These items were taken from two German online surveys, the latter being the largest mental health survey conducted with university students in Germany [28, 29].

The questionnaires have been published in relation to mental health status elsewhere [30].

#### Metadata on consultation reason, referral and characteristics

The metadata were collected as part of the consultation documentation/system records for each consultation. These data included the number and modality of sessions, consultation reason, referral recommendation, language of the consultation (German or English), number of e-mails exchanged, duration of video and chat sessions, and time of day of the interaction. Consultation reasons were recorded using predefined categories and subcategories (Table 1), with an additional free-text option where applicable.

Consultation reasons were classified using predefined operational categories rather than formal clinical diagnoses. Mental health problems referred to specific psychological symptoms or mental health and behavioural difficulties; emotional problems referred primarily to emotional experiences, self-perception, and emotional coping; and psychosocial problems referred to difficulties related to interpersonal, social, academic, occupational, or living circumstances. These categories were used for descriptive purposes and were not considered mutually exclusive clinical diagnoses.

**Table 1:** Categories for consultation reasons

Category	Sub categories
Mental health problems	Exam/presentation anxiety, Phobias/anxiety/panic attacks; Eating behavior, Psychosomatic/physical complaints, Suicidality, Substance use/addictive behavior, Self-harm behavior, Personality disorder/behavioral disorder, Psychotic symptoms, Affective disorders/depression, Trauma/PTSD, Compulsions/OCD, ADHD, other
Emotional problems	Loneliness, Guilt, Self-esteem, Loss/grief, Anger/aggression/impulsivity, Self-doubt, fear of failure, Heartbreak, Other
Psychosocial problems	Crisis, Stress/overload, Family conflicts/problems, Romantic/partner-related conflicts, Friendship conflicts, Work-related conflicts/problems, Bullying/mobbing, Performance/expectation pressure, Sexual harassment/violence, Gender/sexual identity, Discrimination, Migration/refugee experience, Physical suffering (others), Psychological distress (others), Housing/shared apartment situation, Other
University-related problems	Choice of studies/transition into university, Study financing, Conflicts/issues with lecturers, Study organization, Exams, Work-life balance (study & family/children), Studying with disability/impairment, Other
Additional problems	Physical illness/impairment, Procrastination, Time management, Motivational problems, Career prospects after graduation
Not applicable	Not applicable

#### Acceptability and helpfulness outcomes at T1

##### User satisfaction (ZUF-8)

Participant satisfaction was assessed using the ZUF-8 [31] a German adaptation of the Client Satisfaction Questionnaire [32]. The ZUF-8 is available for non-commercial research use. Additionally, participants were asked to answer how they felt after the intervention (answers ranging on a 4-point Likert-scale from 4 = “better”, 3 = “slightly better”, 2 = “slightly worse” to 1 = “worse”) and whether they felt like the intervention had helped them with answers ranging on a 4-point Likert-scale from “yes” to “no”. To measure participants’perceived wellbeing and helpfulness of the consultation, these two self-constructed items were developed by the research team.

Acceptability and helpfulness outcomes were assessed immediately after counselling (invitation via mail).

#### Users vs. Non-Users

Participants who completed the baseline questionnaire and also engaged in additional services, such as any counselling option (email, video, chat), were classified as users. Those who completed the baseline questionnaire but did not take part in any further counselling services (video, chat and/or email) were classified as non-users. Users and non-users were defined regardless the usage of the app.

### Statistical analysis

All statistical analyses were conducted using IBM SPSS Statistics 29. P-values were not adjusted for multiple comparisons. A two-tailed significance level of α = 0.05 was applied to all statistical tests.

Descriptive statistics were calculated for all sociodemographic variables and baseline outcome measures, including the PHQ-9, GAD-7, EDE-Q8, and AUDIT-C. To examine differences between counselling users and non-users, independent-samples t-tests were conducted for continuous variables and chi-square tests for categorical variables, including gender and migration history. Effect sizes for chi-square tests were reported using Cramer’s V. Counselling users were defined as participants who had attended at least one counselling session, irrespective of the counselling modality.

To examine potential predictors of counselling uptake, a binary logistic regression analysis was conducted. Use of the enhance counselling service (0 = no use, 1 = use of at least one counselling session) was entered as the dependent variable. PHQ-9, GAD-7, AUDIT-C, and EDE-Q8 scores, as well as gender and migration history, were included as independent variables.

To assess user satisfaction, data from the ZUF-8 administered immediately after the first counselling session (T1) were analysed. The analysis was restricted to the first session because the number of participants decreased substantially with increasing numbers of sessions. Data from the different counselling modalities were first merged, after which a total ZUF-8 score was calculated for each session. When participants had data from more than one modality, scores were aggregated at the participant level to obtain one mean score per participant. Mean values were also calculated for the additional questions assessing participants’ perceived well-being following the session and whether the counselling had been helpful.

Finally, descriptive analyses were conducted using counselling-session metadata to characterize service utilization. Variables included counselling modality, session language (German or English), appointment time, reason for consultation based on predefined categories (Table 1), and whether a referral recommendation was provided. Baseline questionnaire (T0) data and appointment records were merged and aggregated at the participant level.

On a general note, as no predefined feasibility thresholds were established a priori, feasibility was assessed descriptively based on observed implementation of the counselling service, intervention uptake, and acceptability among users. The final sample comprised 447 participants, including 117 counselling users and 330 non-users. For independent-samples t-tests comparing these two groups, a sensitivity power analysis was conducted assuming a two-tailed significance level of α = 0.05. Given the observed group sizes, the study had approximately 80% statistical power to detect a standardized mean difference of Cohen’s d = 0.302. An effect size of d = 0.30 corresponded to approximately 79.5% power. Accordingly, smaller between-group effects may not have been detected reliably. This analysis relates specifically to continuous between-group comparisons and does not establish the statistical power of the logistic regression or the descriptive analyses of counselling satisfaction and service utilization.

## Results

### Sociodemographic characteristics

A total of *n* = 447 students completed the baseline questionnaire, of whom *n* = 342 (76.5%) identified as female, *n* = 84 (18.8%) as male, and *n* = 21 (4.7%) as diverse (see Table 2). Participants age ranged from 18 to 53 years with a mean age of 24.15 years (*SD* = 4.70). Most participants indicated being single (*n* = 241, 53.9%) and living in a shared accommodation (*n* = 322, 72.0%). Additionally, the majority of participants reported no migration background (*n* = 368,82.3%), and indicated that their parents did not hold an academic degree (*n* = 246, 55.1%; *n* = 222, 49.7%). Roughly half of the participants (*n* = 224, 50.1%) reported being enrolled in a master’s degree program or an equivalent qualification (e.g., state examination qualification or diploma). On average, the participants had been matriculated in their degree program for a duration of 7.91 (*SD* = 4.752) semesters with a range from one to 31 semesters. Furthermore, most participants (*n* = 301, 67.3%) reported having a current monthly net income of less than 1000 €.

**Table 2:** Sociodemographic characteristics of the sample (*n* = 447)

Variable	*n* (%)
**Gender**	
Female	342 (76.5)
Male	84 (18.8)
Diverse	21 (4.7)
**Relationship status**	
Married	15 (3.4)
In a committed relationship	186 (41.6)
In a registered civil partnership	3 (0.7)
Single	241 (53.9)
Divorced	2 (0.4)
**Living situation**	
Alone	125 (28.0)
With partner/spouse, children, other relatives or with others in a shared living arrangement	322 (72.0)
**Targeted academic degree**	
Bachelor	184 (41.2)
Master (or equivalent degree, i.e. state exam or diploma)	224 50.1)
Other	39 (8.7)
**Current monthly net income**	
No personal income	77 (17.2)
0–499 €	81 (18.1)
500–999 €	143 (32.0)
1000–1499 €	106 (23.7)
1500–1999 €	13 (2.9)
2000–2999 €	11 (2.5)
≥ 3000 €	5 (1.1)
No information provided	11 (2.5)
**Migration history**	
Immigrant	42 (9.4)
At least one parent is an immigrant	37 (8.3)
No history of immigration	368 (82.3)
**Highest professional qualification of mother/ first parent**	
No professional qualification	17 (3.8)
Apprenticeship/ vocational training diploma	193 (43.2)
Master craftsman certificate, state certified technician	36 (8.1)
University degree	177 (39.6)
Unknown	7 (1.6)
No information provided	17 (3.8)
**Highest professional qualification of father/ second parent**	
No professional qualification	16 (3.6)
Apprenticeship/ vocational training diploma	138 (30.9)
Master craftsman certificate, state certified technician	68 (15.2)
University degree	194 (43.4)
Unknown	17 (3.8)
No information provided	14 (3.1)

A large proportion of students had not prior engaged with support services regarding their mental health at the time of the study (see Table 3). However, with the exception of seeking help from alternative practitioners specializing in psychotherapy, staying in psychiatric or psychosomatic clinics, or reading self-help books, most participants reported that they were likely or very likely to use support services such as psychiatric or psychotherapeutic treatment and online-based support options. Forms of past utilization of mental health services are presented in Table 4.

**Table 3:** Help-seeking intentions regarding mental health services at baseline (T0), *N* = 447)

What kind of support would you seek if you were experiencing mental health issues?	Very unlikely (*n*, %)	Unlikely (*n*, %)	Likely (*n*, %)	Very likely (*n*, %)
Psychiatrist	61 (13.6)	101 (22.6)	140 (31.3)	145 (32.4)
Psychotherapy	14 (3.1)	41 (9.2)	114 (25.5)	278 (62.2)
Alternative practitioner specializing in psychotherapy	184 (41.2)	132 (29.5)	84 (18.8)	47 (10.5)
Psychological/psychosocial counselling	21 (4.7)	52 (11.6)	152 (33.9)	222 (49.7)
Stay in a psychiatric/psychosomatic clinic	236 (52.8)	116 (25.9)	61 (13.6)	34 (7.6)
Online support services for mental health issues	24 (5.4)	81 (18.1)	148 (33.1)	194 (43.4)
Self-help books	102 (22.8)	128 (28.6)	95 (21.3)	122 (27.3)

**Table 4:** Utilization of mental health services in the past at baseline (T0), *N* = 447)

Which of the following support services for mental health have you used at some point or in the last year?	Already used this service at some point (*n*, %)	Used the service in the past year (*n*, %)	Did not use the service (*n*, %)
Psychiatrist	43 (9.6)	46 (10.3)	358 (80.1)
Psychotherapy	97 (21.7)	84 (18.8)	266 (59.5)
Alternative practitioner specializing in psychotherapy	12 (2.7)	12 (2.7)	423 (94.6)
Psychological/psychosocial counselling	104 (23.3)	77 (17.2)	266 (59.5)
Stay in a psychiatric/psychosomatic clinic	50 (11.2)	12 (2.7)	385 (86.1)
Online support services for mental health issues	65 (14.5)	79 (17.7)	303 (67.8)
Self-help books	84 (18.8)	117 (26.2)	246 (55.0)

Most participants were aware of the Central Student Advisory Service (n = 379, 84.8%) and psychological or psychosocial counselling services (n = 335, 74.9%) available at their universities, while awareness of digital services was lower (n = 164, 36.7%); however, most students had never used these services, particularly digital services (n = 400, 89.5%).

**Table 5:** Knowledge and past utilization of psychosocial counseling and online services at universities (T0), *N* = 447)

To the best of your knowledge, what advisory and support services does your university offer?	Yes (*n*, %)	No (*n*, %)	I do not know (*n*, %)
Central Student Advisory Service	379 (84.8)	3 (0.7)	65 (14.5)
Psychological, psychosocial or psychotherapeutic counselling for students	335 (74.9)	11 (2.5)	101 (22.6)
Support centres for students with children	303 (67.8)	6 (1.3)	138 (30.9)
Digital services	164 (36.7)	22 (4.9)	261 (58.4)
**Which advisory and support services offered by your university have you already made use of?**	**Used this service once**	**Used this service multiple times**	**Never used this service**
Central Student Advisory Service	86 (19.2)	20 (4.5)	341 (76.3)
Psychological, psychosocial or psychotherapeutic counselling for students	78 (17.4)	59 (13.2)	310 (69.4)
Support centres for students with children	10 (2.2)	6 (1.3)	431 (96.4)
Digital services	30 86.79	17 (3.8)	400 (89.5)

### Mental health measures

Participants’ mental health status was assessed using the baseline questionnaire (*n* = 447), with a mean value of 11.85 in the GAD-7 questionnaire (*SD* = 4,66), 12.64 in the PHQ-9 (*SD* = 5.72) and 15.31 in the EDE-Q8 (*SD* = 12.68). The Audit-C questionnaire was answered by *n* = 349 participants, with the remaining 98 participants stating not consuming alcohol at all. The mean value was 3.69 (*SD* = 2.38).

#### Discrimination

A total of *n* = 63 (14.1%) participants answered the baseline questionnaire including the additional items addressing experiences of discrimination. The majority of students stated that they did not have an impairment (*n* = 44, 69,8%) or a disability (*n* = 57, 90,5%). Most participants report that they did not have experiences of discrimination based on disabilities or chronic illnesses (*n* = 53, 84,1%), racism or nationality (*n* = 57, 90,5%), gender (*n* = 36, 57,1%), gender identity (*n* = 59, 93,7%), religion or beliefs (*n* = 58, 92,1%), advanced (*n* = 58, 92,1%) or young age (*n* = 57, 92,1%), sexual orientation (*n* = 53, 90,5%), low educational attainment (*n* = 57, 84,1%) and low income (*n* = 53, 84,1%).

### Metadata on consultation reason, referral and users characteristics

Video counselling was the most frequently used modality for the first consultation (*n* = 75, 41.0%), followed by chat (*n* = 34, 18.0%) and e-mail counselling (*n* = 28, 15.3%).

The mean value of sessions attended per person was 1.30 (*SD* = 0.53) with a minimum of one and a maximum of four sessions. The majority of the consultations were conducted in German (*n* = 104, 89.7%) with *n* = 11 sessions (10.4%) taking place in English. Reasons for the consultations were mental health concerns (*n* = 108, 60.0%), followed by emotional (*n* = 20, 11.1%) and psychological concerns (*n* = 19, 10.6%). The remaining *n* = 33 sessions (18.3%) addressed other reasons, including university-related and additional problems, or were not categorised (Table 1). Out of *n* = 183 sessions in total, referral recommendations to appropriate care or support services were provided in *n* = 148 (80.9%) cases. These included further enhance sessions (*n* = 65, 43.9%), outpatient psychotherapy (*n* = 30, 20.3%), psychosocial counselling centres (*n* = 29, 19.6%), consultations with a doctor or physician (*n* = 10, 6.8%), app usage (*n* = 5, 3.4%), specialised counselling centres (*n* = 4, 2.7%), university counselling centres (*n* = 3, 2.0%), emergency services (*n* = 1, 0.7%), and other services (*n* = 1, 0.7%). In the remaining cases, no additional referral or support recommendation was considered necessary.

The chat or video conversations had an average duration of *M* = 29.97 min (*SD* = 3,94; Min = 15.00, Max = 40.00) and were scheduled mostly in the afternoon ranging from 12:00 to 18:00 (*n* = 91, 58.7%).

The Mental Health App was downloaded 343 times.

### Acceptability and feasibility outcomes

A total of *n* = 51 participants completed the questionnaire immediately following the first counselling session. User satisfaction was assessed using the ZUF-8, yielding a mean score of 20.14 (*SD* = 1.66). Out of *n* = 49 participants, 47 (95,9%) felt better/ slightly better after the intervention (“better”: *n* = 11, 22,4%; “slightly better”: *n* = 36; 73,5%; “slightly worse”: *n* = 2, 3,9%). Out of *n* = 51 participants, 40 (78,4%) indicated that the offered services were of help to them (“yes”: *n* = 12, 23,5%; “rather yes”: *n* = 28, 54,9%; “rather no”: *n* = 10, 19,6%; “no”: *n* = 1, 2,0%).

### Users vs. non-users

A total of *n* = 117 participants attended at least one video, chat or mail consultation.

Users and non-users did not differ significantly in age, gender, mental health (assessed through the PHQ-9, GAD-7, EDE-Q8 and AUDIT-C) or migration history, as shown in Tables 6 and 7.

The mean age was comparable between users (M = 24.11, SD = 4.58) and non-users (M = 24.28, SD = 5.05), t(187.69) = -0.326, *p* = 0.745, 95% CI [-1.220, 0.874], with a negligible effect size (Hedges’ g = -0.037). Similarly, no significant differences in gender distribution were observed between users and non-users, χ²(2) = 0.705, *p* = 0.703, Cramer’s V = 0.040. Among users, 78.6% identified as female, 17.9% as male, and 3.4% as diverse, compared to 75.8%, 19.1%, and 5.2% among non-users, respectively (see Table 6).

The logistic regression model including all predictors significantly improved the prediction of help-seeking behavior compared to the null model ($\:{\chi\:}^{2}$ = 19.73, *p* = 0.006), explaining 8% of the variance (Nagelkerke $\:{R}^{2}$= 0.08). The regression showed that higher levels of eating disorder symptoms (EDE-Q8) were associated with a significantly lower likelihood of seeking help (B = -0.022, SE = 0.01, *p* = 0.04, OR = 0.98). Additionally, participants with at least one parent with a migration history were significantly less likely to seek help (B = -1.32, SE = 0.63, *p* = 0.038, OR = 0.27). Other variables, including depressive symptoms (PHQ-9), anxiety symptoms (GAD-7), alcohol use (AUDIT-C), gender and personal migration background all assessed at baseline (T0), were not significant predictors of help-seeking behaviour (Table 6).

A second logistic regression examined wether intentions to seek different forms of professional support predicted help-seeking behaviour (Table 3). The model did not significantly predict help-seeking behaviour ($\:{\chi\:}^{2}$= 2.12, *p* = 0.953, *df* = 7), explaining less than 1% of the variance (Nagelkerke $\:{R}^{2}$= 0.007). None of the predictors reached statistical significance. The third regression model investigated predictors related to the utilization of mental health services in the past (Table 4). The set of predictors did not significantly explain help-seeking behaviour ($\:{\chi\:}^{2}$= 16.96, *p* = 0.258, *df* = 14 Nagelkerke $\:{R}^{2}$ = 0.054). Awareness of available university support services (Table 5) was not significantly associated with help-seeking behaviour, as indicated by a non-significant logistic regression model ($\:{\chi\:}^{2}$= 7.94, *p* = 0.440, *df* = 8; Nagelkerke $\:{R}^{2}$ = 0.026). No significant predictors emerged. The regression model examining prior utilization of university support services did not significantly predict help-seeking behaviour($\:{\chi\:}^{2}$= 8.56, *p* = 0.38, *df* = 8; Nagelkerke $\:{R}^{2}$ = 0.028). Within this model, students who had used digital support services once had significantly lower odds of help-seeking behavior than those who had never used these services (B = -1.38, SE = 0.64, *p* = 0.031, *df* = 1, OR = 0.25). However, the overall model was not statistically significant.

**Table 6:** Predictors of Help-Seeking Behavior (usage vs. non-usage), *N* = 447

PHQ-9	B	SD	df	*p*	OR
	0.01	0.03	1	0.67	1.01
GAD-7	0.07	0.04	1	0.06	1.07
EDE-Q8	-0.02	0.01	1	0.04	0.98
AUDIT-C	-0.07	0.06	1	0.24	0.94
Immigrant	0.36	0.41	1	0.39	1.43
At least one parent is an immigrant	-1.32	0.63	1	0.04	0.27
Gender	-0.38	0.26	1	0.15	0.69

**Table 7:** Sociodemodraphic differences between Users and Non-Users, *N* = 447

Variable, *n* (%)	User (*n* = 117)	Non-User (*n* = 330)	Pearson’s Chi square	df	*p*	Cramers’s V	
**Gender**, *n* (%)			0.71	2	0.70	0.04	
Female	92 (78.6)	250 (75.8)					
Male	21 (17.9)	63 (19.1)					
Diverse	4 (3.4)	17 (5.2)					
**Background of migration**, *n* (%)			3.64	2	0.16	0.09	
Immigrant	16 (13.7)	26 (7.9)					
At least one parent is an immigrant	8 (6.8)	29 (8.8)					
No history of migration	93 (79.5)	275 (83.3)					

**Table 8:** Differences between Users and Non-Users in the mental health measures, *N* = 447

Variable (Mental Health Outcomes)	User (*n* = 117)	Non-User (*n* = 330)				95% Confidence Interval		
			T	df	*p*	Lower limit	Upper limit	Effect size (Hedges)
PHQ-9, *M* (*SD*)	13.03 (5.82)	12.49(5.69)	-0.87	199.89	0.39	-1.77	0.69	-0.09
GAD-7, *M* (*SD*)	12.39 (4.82)	11.66 (4.59)	-1.42	195.44	0.16	-1.74	0.28	-0.16
EDE-Q8, *M* (*SD*)	13.75 (11.37)	15.85 (13.08)	1.65	232.37	0.10	-0.41	4.62	0.17
	User (*n* = 92)	Non-User (*n =* 257*)*						
*AUDIT-C, *M* (*SD*)	3.39 (2.27)	3.81 (2.41)	1.49	169.43	0.14	-0.14	0.97	0.18

* were calculated using valid cases because of missing data

## Discussion

This study aimed to evaluate the feasibility and acceptability of the digital mental health platform “enhance”, a low-threshold support service availale for six universities in Saxony, Germany. This study particularly focused on the psychological counselling service delivered by trained psychologists or psychotherapists in training to university students by via chat, video, and/or email. Counselling users were characterized and differences between users and non-users regarding sociodemographic variables and mental health outcomes were examined. Participants filled standardized assessments at baseline (T0) and post-intervention (T1). Overall, 447 students completed baseline assessment, with results indicating moderate anxiety. Of these, 117 engaged in at least one counselling session, with video counselling being the main option of choice (41%). Although only 26% of participants who completed the baseline questionnaire engaged with at least one counselling session (therefore classified as users for the purpose of this study), satisfaction was high (ZUF-8: M = 20.14), and 95.9% of participants reported improved well-being after sessions, and 78.4% found the intervention helpful at post-intervention assessment.

The present findings suggest that the enhance program is a feasible and well-accepted low-threshold mental health support service for university students. Overall, participants reported high levels of satisfaction and perceived helpfulness, while the counselling sessions appeared to function effectively both as short-term support and as a gateway to further mental health care through targeted referral recommendations.

The sample was predominantly female, with a mean age of 24 years. A substantial proportion of participants reported relatively low monthly income and non-academic parental backgrounds, suggesting that many students may face socioeconomic challenges during their studies, as well was that they might be the first generation to achieve a higher education degree. Previous research indicates that financial strain and academic pressure can contribute to psychological distress among university students and may increase vulnerability to mental health issues [33].

Concerning experiences of discrimination, the majority of participants reported no experiences related to disability, ethnicity, religion, or sexual orientation. Prior research suggests that perceived discrimination and minority stress can negatively impact psychological well-being and may contribute to increased anxiety and depressive symptoms in student populations, highlighting the importance of inclusive campus environments and diversity-sensitive support services [30]. The baseline mental health assessment revealed comparatively high levels of psychological distress among participants enrolled in the platform, with mean scores on the GAD-7 and PHQ-9 indicating moderate symptoms of anxiety and depression. These findings are consistent with a growing body of literature demonstrating elevated levels of mental health problems among university students worldwide, with systematic reviews estimating approximately 40% of university students experience clinically relevant anxiety symptoms [34], and having remained as a at-risk group far beyond the pandemic [35].

Findings indicated high level of acceptance of professional mental health support among students enrolled in the platform. Psychotherapy, psychological counselling, and online support services were identified as the preferred options for addressing mental health concerns, whereas inpatient psychiatric treatment and support from alternative psychotherapy practitioners were viewed less favorably. This pattern suggests a preference for low-threshold, outpatient, and easily accessible forms of mental health care. In terms of prior help-seeking behavior, psychotherapy, psychosocial counselling, self-help books, and online services were among the most commonly utilized mental health resources.

The comparatively high use of digital support services suggests that online mental health interventions represent an important and increasingly relevant source of support for university students in this sample. Most participants were aware of central student advisory services (*n* = 379, 84.8%) and psychological counselling (*n* = 335, 74.9%) offered by their universities, whereas awareness of digital services was substantially lower. Despite this general awareness, the majority of students reported never having used university support services, depending on the type of service (69.4% – 94.6%). This discrepancy between general awareness of help services, positive attitudes toward help-seeking, and actual (= low) service utilization may reflect barriers such as stigma, limited visibility of available services, or restricted accessibility [9]. Taken together, the baseline findings describe a student population experiencing considerable psychological distress alongside common socioeconomic challenges associated with university life. Overall, the results underline the importance of accessible, well-communicated, and low-threshold mental health support services within university settings, particularly in digital formats.

In terms of feasibility, a total of *n* = 117 (individuals attended at least one consultation (from originally *N* = 447), with most participants attending a single session. This usage pattern is consistent with other brief support services and walk-in or single-session counselling models, designed to address immediate concerns and facilitate further help-seeking if necessary [36]. At the same time, a considerable proportion of participants (76.7%) had already downloaded and used the app and may have been sufficiently supported by the digital content alone, potentially reducing the perceived need for additional counselling sessions.

Afternoon appointment slots were particularly popular, which may reflect students’ academic schedules and daily routines. These findings suggest that flexible scheduling and the provision of multiple communication formats, such as video, chat, and e-mail counselling, may forster both accessibility and engagement with mental health support services.

Overall, the counselling sessions demonstrated a high level of acceptability among participating students, with video counselling emerging as the preferred modality. Satisfaction with the service, assessed using the ZUF-8, was high (*M* = 20.14), indicating that participants evaluated the programme positively. These findings are in line with previous research on brief and digital mental health interventions for university students, which similarly reports high levels of satisfaction and perceived usefulness for low-threshold support services [11, 16, 36].

These findings highlight the importance of accessible and flexible support formats for students experiencing psychological distress. Consistent with the high satisfaction ratings, most participants reported subjective improvements in well-being following their session, with nearly all respondents indicating that they felt at least slightly better after a chat or video consultation. This suggests that even a single session may provide meaningful short-term support, in line with previous research on brief and low-intensity interventions in university settings [16, 36]. Perceived helpfulness of the service was also high, further supporting the value of timely and easily accessible mental health support for students.

Importantly, the majority of sessions (80.3%) resulted in referral recommendations to additional care or support services, suggesting that enhance may serve as an effective gateway to more specialized mental health care. Furthermore, most consultations focused on mental health-related concerns, underlining both the relevance of the programme and the substantial psychological burden experienced by university students [5, 9].

Overall, users and non-users showed few differences regarding sociodemographic characteristics and mental health outcomes. However, participants with higher eating disorder symptomatology were less likely to engage with the counselling services, consistent with previous findings on lower engagement in digital interventions among individuals with more severe symptoms [37]. Similarly, participants with a parental migration history were less likely to seek help, potentially reflecting cultural, linguistic, or structural barriers [38]. Future iterations should therefore consider targeted, culturally sensitive, and accessible approaches to reduce potential barriers to engagement. However, the underlying reasons for these associations remain unclear and should be investigated in future research.

In contrast, depressive symptoms, anxiety symptoms, alcohol use, gender, and personal migration background were not significantly associated with service utilization. Help-seeking intentions and prior use of help-seeking did also not predict engagement with the service. Together, these findings suggest that the intervention may be applicable to a broad range of students experiencing mental health difficulties.

Future research should also incorporate qualitative approaches to better understand the factors influencing engagement and retention. Interviews or focus groups with both users and non-users could provide insight into perceived barriers and facilitators, including usability, accessibility, perceived relevance, privacy concerns, time constraints, and preferences regarding digital versus face-to-face support. Qualitative research may be particularly valuable for exploring the reasons underlying the lower engagement observed among students with higher eating disorder symptomatology and those with a parental migration history. Understanding these experiences could inform the further tailoring of the intervention and support the development of strategies to improve sustained engagement and retention.

## Limitations

Some limitations of the present study should be considered when interpreting the findings. First, the voluntary nature of participation may have introduced self-selection bias, as individuals engaging with digital mental health interventions may differ from non-participants in help-seeking attitudes, digital and mental health literacy, or symptom awareness. Second, the absence of a randomized controlled design limits causal conclusions regarding intervention effectiveness. Third, the reliance on self-report measures may have increased the risk of reporting bias, including social desirability effects and inaccuracies in symptom reporting. Fourth, the conclusions taken from the post-intervention assessment limit the long-term sustainability of the observed effects. Among students who engaged with the programme, counselling sessions were associated with high satisfaction ratings and self-reported short-term perceived improvements in well-being. These findings provide preliminary evidence regarding the acceptability and observed implementation of the programme but do not establish its effectiveness. Although counsellors provided referral recommendations when specialised care was considered appropriate, the present study did not assess whether students subsequently accessed or engaged with the recommended services. Additionally, the relatively small number of intervention users compared to non-users may have reduced statistical power for subgroup analyses and predictors of help-seeking behavior. Finally, although participants were recruited from multiple universities in Saxony, the geographically and culturally specific sample may limit the generalizability of the findings to other contexts. Non-response and missing data represent another potential limitation of the study. Participants who did not complete the T1 questionnaire, or who provided incomplete responses to T1, may have differed systematically from those who provided complete data. For example, participants who remained engaged with the intervention may have been more likely to complete T1 asessment. Such differences could have introduced non-response or attrition bias and may limit the generalisability of the findings. Although the number of available observations was considered in the analyses, the possibility of bias due to unobserved differences between users and non-users cannot be completely excluded. Furthermore, the mental health and behavioural outcomes were assessed using self-report questionnaires and may therefore be subject to recall or social desirability bias. The consultation metadata were derived from routine consultation records and predefined categories, which may not fully capture the complexity of participants’ reasons for seeking support. In addition, consultation-related metadata were available only for participants who engaged with the respective services and therefore cannot be assumed to represent the entire baseline sample.

## Conclusion

Enhance is an online programme designed to provide personalized support for university students experiencing mental health difficulties by offering short-term counselling and facilitating referrals to specialized care when needed. To our knowledge, it is among the first student-specific systems combining an app-based platform with individualized counselling via email, chat, and video sessions in Germany. In addition to providing practical support, the programme may also offer students a safe and supportive space in which they feel heard, validated, and guided according to their individual needs.

Overall, the findings indicate that the programme was feasible to implement and was well received among students who engaged with the intervention, as reflected by high satisfaction ratings, perceived improvements in well-being, and successful implementation of counselling sessions. However, uptake was limited, with 117 of 447 participants engaging with the digital intervention. In addition, higher eating disorder symptomatology and parental migration history were associated with lower engagement, highlighting the need for further investigation of potential barriers to access and use. Future iterations could incorporate more targeted and culturally sensitive approaches to improve accessibility and engagement, including tailored information and support for students with eating-related concerns and students from diverse migration backgrounds. Qualitative research with both users and non-users could further elucidate barriers and facilitators influencing engagement and retention. Further research is also needed to examine the longer-term effects of the programme and whether adaptations designed to improve engagement translate into sustained use and improved mental health outcomes.

## Supplementary Information

Below is the link to the electronic supplementary material.

Supplementary Material 1

Supplementary Material 2

Supplementary Material 3

Supplementary Material 4

## Data Availability

The data that support the findings of this study are not openly available due to reasons of sensitivity and are available from the corresponding author upon reasonable request. Data are located in controlled access data storage at Leipzig University, Medical Faculty.
