# Supplementary material for: Feasibility, acceptance, user satisfaction, and differences between users and non-users of an online-based support service for University students
Source: Discov Ment Health. 2026 Nov 11;6(1):223. doi: 10.1007/s44192-026-00648-5 (PMC13755097; doi:10.1007/s44192-026-00648-5)
Supplement: Supplementary file 1 [file 44192_2026_648_MOESM1_ESM.pdf]

Fragebogen

1 Soziodemographie

Sociodemographic Information

At which university do you study?

Please use the free text field for your entry.

Are you female, male, or diverse?

- ◯ Female
- ◯ Male
- ◯ Diverse

How old are you?

(in years)

What is your marital status?

- ◯ Married
- ◯ In a relationship
- ◯ Registered civil partnership
- ◯ Single
- ◯ Divorced

Do you live alone or together with other people in a shared household?

- ◯ Alone
- ◯ With partner/spouse, children, other relatives or persons, or in a shared living arrangement

Are you studying for a Bachelor's degree, Master's degree, or another type of degree?

- ◯ Bachelor's degree
- ◯ Master's degree (or equivalent, e.g. state examination, diploma)
- ◯ Others, specifically

In total, across all degree programs, for how many semesters have you been enrolled at university?

Please answer only in whole numbers (e.g., "6" or "12")

What is your current monthly net income?

(Note: Please refer to the sum of all your personal financial income, i.e., your income as an individual.)

- ◯ No personal income
- ◯ 0 - 499 €
- ◯ 500 - 999 €
- ◯ 1000 - 1499 €
- ◯ 1500 - 1999 €
- ◯ 2000 - 2999 €
- ◯ 3000 and above
- ◯ No information

Do you have a migration background?*(Multiple answers possible)*

- ☐ Yes, I am an immigrant myself
- ☐ Yes, at least one of my parents is an immigrant
- ☐ No

What is the highest professional qualification of your mother or your first parent?

- ◯ No vocational qualification
- ◯ Apprenticeship or skilled worker qualification
- ◯ Master craftsman's diploma, technical college degree
- ◯ University degree
- ◯ Unknown
- ◯ Prefer not to say

What is the highest professional qualification of your father or your second parent? ?

- ◯ No vocational qualification
- ◯ Apprenticeship or skilled worker qualification
- ◯ Master craftsman's diploma, technical college degree
- ◯ University degree
- ◯ Unknown
- ◯ Prefer not to say

2 Beeinträchtigung ja/nein

Do you have a disability that makes studying difficult?

- ◯ Yes
- ◯ No

3.1 Filter Beeinträchtigung

If so, which one

- ◯ Mental illness (e.g. depression, eating disorder)
- ◯ Long-term/chronic physical illness (e.g. rheumatism, multiple sclerosis, intestinal disease)
- ◯ Specific learning disability (e.g. dyslexia, dyscalculia)
- ◯ Movement impairment (e.g. limitations when walking, standing, grasping)
- ◯ Visual impairment or blindness (e.g. limitations in visual perception with regard to visual acuity, field of vision or colour perception)
- ◯ Hearing impairment or deafness (e.g. reduced hearing ability, hearing loss)
- ◯ Multiple impairments of equal severity (more than one impairment that hinders study, none of which clearly dominates)
- ◯ Other impairments or illnesses (e.g. speech impairment, stuttering, tumours, autism spectrum disorder, ADHD, migraine)

4 Grad der Behinderung

Have you been assessed as having a degree of disability (GdB)?

- Yes
- No

5.1 Filter GdB

If so, what is your degree of disability?

- 20
- 30
- 40
- 50
- 60
- 70
- 80
- 90

6 Diskriminierungserfahrungen

Experiences of discrimination

The next topic is discrimination. Discrimination means that a person is treated worse than other people for certain reasons, without there being any objective justification. Discrimination can be practiced in very different ways, for example through insults, exclusion, sexual harassment and even violence. However, discrimination also occurs when people are disadvantaged by rules and laws. Have you personally been discriminated against in Germany in the last 24 months for the following reasons?

	Yes	No	No information
For racist reasons, because of membership of an ethnic group or origin from another country	◯	◯	◯
On the basis of gender	◯	◯	◯
On the basis of gender identity (e.g. transgender or intersex)	◯	◯	◯
On grounds of religion or belief	◯	◯	◯
Due to disability or chronic illness	◯	◯	◯
Due to old age	◯	◯	◯
Due to being too young	◯	◯	◯
Due to sexual orientation (e.g. gay, lesbian, bisexual)	◯	◯	◯
Due to a low level of education	◯	◯	◯
Due to low income	◯	◯	◯
For another reason	◯	◯	◯

If you selected ‘for another reason’, what was it?

7 Psychische Gesundheit 1

Mental Health

Health Questionnaire

Over the *last 2 weeks,* *how often have you been bothered by the following problems??*

	Not at all	Several days	More than half the days	Nearly every day
1. Feeling nervous, anxious or on edge	◯	◯	◯	◯
2. Not being able to stop or control worrying	◯	◯	◯	◯
3. Worrying too much about different things	◯	◯	◯	◯
4. Trouble relaxing	◯	◯	◯	◯
5. Being so restless that it is hard to sit still	◯	◯	◯	◯
6. Becoming easily annoyed or irritable	◯	◯	◯	◯
7. Feeling afraid as if something awful might happen	◯	◯	◯	◯

Current well-being

Over the last 2 weeks, how often have you been bothered by the following problems?

	Not at all	Several days	More than half the days	Nearly every day
1. Little interest or pleasure in doing things	○	○	○	○
2. Feeling down, depressed, or hopeless	○	○	○	○
3. Trouble falling or staying asleep, or sleeping too much	○	○	○	○
4. Feeling tired or having little energy	○	○	○	○
5. Poor appetite or overeating	○	○	○	○
6. Feeling bad about yourself or that you are a failure or have let yourself or your family down	○	○	○	○
7. Trouble concentrating on things, such as reading the newspaper or watching television	○	○	○	○
8. Moving or speaking so slowly that other people could have noticed. Or the opposite being so fidgety or restless that you have been moving around a lot more than usual	○	○	○	○
9. Thoughts that you would be better off dead, or of hurting yourself	○	○	○	○

7.1.1 Hinweis Suizidalität

We have already explained to you that all responses to this survey are stored anonymously. In this questionnaire, you have indicated that you have thoughts of death or of doing something to yourself at least on several days (or more frequently).

*If you fear harming yourself or have thoughts of death **it is important to seek professional help.***

If you are in an acute crisis, please dial 112 or go to the nearest hospital (e.g., also accompanied by a trusted person)!

You can also use the following contact points: # Psychosocial student counseling of Leipzig University # Telephone helplines: 0800 111 0 111 - 0800 111 0 222 - 116 123

Please confirm that you have taken note of the information

○ *I have read and taken note of the information*

8 Psychische Gesundheit 2

Alcohol Consumption

	Never	Monthly or less	2-4 times a month	2-3 times a week	4 or more times aweek
How often do you have a drink containing alcohol?	○	○	○	○	○

Alcohol Consumption

	1 or 2	3 or 4	5 or 6	7 to 9	10 or more	I do not drink alcohol
How many standard drinks containing alcohol do you have on a typical day when drinking? (One glass of alcohol corresponds to: 0.33 l beer, 0.25 l wine or sparkling wine, 0.02 l spirit.)	○	○	○	○	○	○

Alcohol Consumption

	Never	Less than monthly	Monthly	Weekly	Daily or almost daily
How often do you have 6 or more drinks on one occasion? (One glass of alcohol corresponds to: 0.33 l beer, 0.25 l wine or sparkling wine, 0.02 l spirit.)	○	○	○	○	○

Drug/Substance Use

	Never	Monthly or less	2-3 times a month	2-4 times a week	4 or more times a week
How often do you use drugs/substances?	○	○	○	○	○

Body image and Eating behavior

Over the last 4 weeks (28 days)...

	Never		Rarely		Sometimes		Often
1. How dissatisfied were you with your weight?	○	○	○	○	○	○	○
2. How uncomfortable did you feel when you saw your body (e.g., in the mirror, your reflec-tion in a shop window, undressing, bathing, or showering)?	○	○	○	○	○	○	○

Body image and Eating behavior

Over the last 4 weeks (28 days)...

	Not at all	Several times	Less than half the time	Half the time	More than half the time	Nearly every time	Every time
3. In how many of the situations, in which you ate, did you feel guilty (e.g., feel like to did something wrong), because of the impact on your physique or weight (Please do not count binge eating)?	○	○	○	○	○	○	○

Body image and Eating behavior

Over the last 4 weeks (28 days)...

	Not at all	1-5 day(s)	6-12 days	13-15 days	16-22 days	23-27 days	Every day
4. Have you consciously tried to limit the amount of food you eat to affect your physique or weight (regardless of whether you actually succeeded)?	○	○	○	○	○	○	○
5. Have you tried to exclude foods you like from your diet to affect your physique or weight (regardless of whether you actually succeeded)?	○	○	○	○	○	○	○
6. Has thinking about über food, eating, or calories made it difficult for you to focus on things that interest you (e.g., working, following a conversation, or reading)	○	○	○	○	○	○	○
7. Did you feel fat	○	○	○	○	○	○	○
8. Did you have a strong desire to lose weight?	○	○	○	○	○	○	○

9 Nutzung von Hilfsangeboten

Use of help-services

What kind of services would you use if you had mental health problems?

	Very unlikely			Very likely
Psychiatrist	○	○	○	○
Psychotherapist	○	○	○	○
Alternative/non-medical practitioner for psychotherapy	○	○	○	○
Psychological/psychosocial Counselling	○	○	○	○
Stay in a psychiatric/psychosomatic clinic	○	○	○	○
Online help services for mental health problems (e.g., Apps, self-management programs)	○	○	○	○
Self-help books/guides	○	○	○	○
Other: ____	○	○	○	○

Which of the following services for help with mental health problems have you used before or in the last year? *(Multiple answers possible)*

	I have already made use of it	I have made use of it in the last year	Not applicable
Psychiatrist	○	○	○
Psychotherapy	○	○	○
Alternative/non-medical practitioner for psychotherapy	○	○	○
Psychological/psychosocial Counselling	○	○	○
Stay in a psychiatric/psychosomatic clinic	○	○	○
Online help services for mental health problems (e.g., Apps, self-management programs)	○	○	○
Self-help books/guides	○	○	○
Other: ________	○	○	○

To your knowledge, what services does your university offer for coping with stress and stress management?

	Yes	No	I don't know
Central Student Advisory Service	○	○	○
Psychological/psychosocial/psychotherapeutic Counselling for Students	○	○	○
Counselling services for students with children	○	○	○
Digital/online services	○	○	○
Other: ________	○	○	○

Which services for coping with stress and stress management at your university have you al-ready used?

	I used it once	I used it multiple times	Never
Central Student Advisory Service	○	○	○
Psychological/psychosocial/psychotherapeutic Counselling for Students	○	○	○
Beratungsstellen für Studierende mit Kind	○	○	○
Counselling services for students with children	○	○	○
Other: ________	○	○	○

10 Gewissenhafte Bearbeitung

Conscientious completion of the questionnaire is important to ensure the study's validity.

You will not suffer any disadvantages, regardless of which answer you choose. Your participation in the voucher lottery and your chance to win will also remain unaffected by your answer. This question only serves to ensure the quality of the data.

- ◯ No, I did not complete the questionnaire conscientiously and with due diligence.
- ◯ Yes, I completed the questionnaire conscientiously and with due diligence.

11 Angebot_erfahren

We would be interested to know how you found out about our offer.

- ◯ Email from an email distribution list
- ◯ Newsletter of an organisation, initiative, association, etc.
- ◯ Social media post
- ◯ Flyers/postcards
- ◯ Posters
- ◯ Recommendation from friends or family members
- ◯ Recommendation by other support services
- ◯ Others

12 Endseite

Vielen Dank für Ihre Teilnahme!

Einige Fragen bezogen sich direkt auf Ihre psychische Gesundheit. Wenn es Ihnen nicht gut geht, Sie Gesprächsbedarf oder ein Anliegen haben, möchten wir Sie dazu einladen, unser Unterstützungsangebot zu nutzen.

Wenn Sie sich jedoch in einer akuten Krise befinden, wählen Sie bitte die 112 oder begeben Sie sich (z. B. auch in Begleitung einer vertrauten Person) in das nächste Krankenhaus!
