# Supplementary material for: Feasibility, acceptance, user satisfaction, and differences between users and non-users of an online-based support service for University students
Source: Discov Ment Health. 2026 Nov 11;6(1):223. doi: 10.1007/s44192-026-00648-5 (PMC13755097; doi:10.1007/s44192-026-00648-5)
Supplement: Supplementary file 2 [file 44192_2026_648_MOESM2_ESM.pdf]

Fragebogen

1 Soziodemographie

Soziodemographische Angaben

An welcher Hochschule studieren Sie?

Bitte nutzen Sie das Freitextfeld für Ihre Eingabe

Sind Sie weiblich, männlich oder divers?

- ○ Weiblich
- ○ Männlich
- ○ Divers

Wie alt sind Sie?

(in Jahren)

Welchen Familienstand haben Sie?

- ○ Verheiratet
- ○ In fester Partnerschaft
- ○ Eingetragene Lebenspartnerschaft
- ○ Alleinstehend
- ○ Geschieden

Leben Sie allein oder zusammen mit anderen Personen in häuslicher Gemeinschaft?

- ○ Allein
- ○ Mit Partner:in/Ehepartner:in, Kindern, anderen Angehörigen oder Personen oder in einer Wohngemeinschaft

Studieren Sie im Bachelor, Master oder in einem anderen Studium?

- ◯ Bachelor
- ◯ Master (oder Äquivalent, z.B. Staatsexamen, Diplom)
- ◯ Anderes, und zwar ______

Wie viele Semester sind Sie insgesamt, über alle Studiengänge hinweg, immatrikuliert?

Bitte antworten Sie nur in ganzen Zahlen (z. B. "6" oder "12")

______

Wie hoch ist Ihr aktuelles monatliches Nettoeinkommen?

(Hinweis: Bitte beziehen Sie sich auf die Summe aller Ihrer persönlichen finanziellen Einkünfte, d. h. Ihrer Einkünfte als einzelne Person.)

- ◯ Kein eigenes Einkommen
- ◯ 0 - 499 €
- ◯ 500 - 999 €
- ◯ 1000 - 1499 €
- ◯ 1500 - 1999 €
- ◯ 2000 - 2999 €
- ◯ 3000 und mehr
- ◯ Keine Angabe

Haben Sie einen Migrationshintergrund? *(Mehrfachnennungen möglich)*

- ☐ Ja, ich bin selbst Immigrant:in / Einwanderer:in
- ☐ Ja, mindestens einer meiner Eltern ist Immigrant:in / Einwanderer:in
- ☐ Nein

Was ist der höchste berufliche Abschluss Ihrer Mutter bzw. Ihres 1. Elternteils?

- ◯ Kein Berufsabschluss
- ◯ Lehre bzw. Facharbeiterabschluss
- ◯ Meisterprüfung, Fachschul-/ Technikerabschluss
- ◯ Hochschulabschluss
- ◯ unbekannt
- ◯ Keine Angabe

Was ist der höchste berufliche Abschluss Ihres Vaters bzw. Ihres 2. Elternteils?

- ◯ Kein Berufsabschluss
- ◯ Lehre bzw. Facharbeiterabschluss
- ◯ Meisterprüfung, Fachschul-/ Technikerabschluss
- ◯ Hochschulabschluss
- ◯ unbekannt
- ◯ Keine Angabe

2 Beeinträchtigung ja/nein

Liegt bei Ihnen eine studienerschwerende Beeinträchtigung vor?

- ◯ Ja
- ◯ Nein

3.1 Filter Beeinträchtigung

Wenn ja, welche?

- ◯ Psychische Erkrankung (z.B. Depression, Essstörung)
- ◯ Körperlich länger andauernde/chronische Erkrankung (z.B. Rheuma, Multiple Sklerose, Darmerkrankung)
- ◯ Teilleistungsstörung (z.B. Legasthenie, Dyskalkulie)
- ◯ Bewegungsbeeinträchtigung (z.B. Einschränkungen beim Gehen, Stehen, Greifen)
- ◯ Sehbeeinträchtigung oder Blindheit (z.B. Einschränkungen der visuellen Wahrnehmungsfähigkeit hinsichtlich der Sehschärfe, des Gesichtsfeldes oder der Farbwahrnehmung)
- ◯ Hörbeeinträchtigung oder Gehörlosigkeit (z.B. Minderungen des Hörvermögens, Schwerhörigkeit)
- ◯ Gleich schwere Mehrfachbeeinträchtigung (Mehr als eine studienerschwerende Beeinträchtigung, von der keine klar dominiert)
- ◯ Andere Beeinträchtigung bzw. Erkrankung (z.B. Sprechbeeinträchtigung, Stottern, Tumorerkrankungen, Autismus-Spektrum-Störung, AD(H)S), Migräne)

4 Grad der Behinderung

Wurde bei Ihnen ein Grad der Behinderung (GdB) festgestellt?

- ◯ Ja
- ◯ Nein

5.1 Filter GdB

Wenn ja, wie hoch ist Ihr Grad der Behinderung?

- ◯ 20
- ◯ 30
- ◯ 40
- ◯ 50
- ◯ 60
- ◯ 70
- ◯ 80
- ◯ 90

6 Diskriminierungserfahrungen

Diskriminierungserfahrungen

Nun geht es um das Thema Diskriminierung. Diskriminierung bedeutet, dass eine Person aus bestimmten Gründen schlechter behandelt wird als andere Menschen, ohne dass es dafür eine sachliche Rechtfertigung gibt. Diskriminierung kann auf sehr unterschiedliche Weise ausgeübt werden, etwa durch Beleidigung, Ausgrenzung, sexuelle Belästigung bis hin zu Gewalt. Es handelt sich aber auch um Diskriminierung, wenn Menschen durch Regeln und Gesetze benachteiligt werden.

Sind Sie persönlich in den letzten 24 Monaten in Deutschland aus den folgenden Gründen diskriminiert worden?

	Ja	Nein	Keine Angabe
Aus rassistischen Gründen, wegen der Zugehörigkeit zu einer ethnischen Gruppe oder der Herkunft aus einem anderen Land	○	○	○
Aufgrund des Geschlechts	○	○	○
Aufgrund der Geschlechtsidentität (z.B. transsexuell oder intergeschlechtlich)	○	○	○
Aufgrund der Religion oder Weltanschauung	○	○	○
Aufgrund einer Behinderung oder chronischen Krankheit	○	○	○
Aufgrund zu hohen Alters	○	○	○
Aufgrund zu niedrigen Alters	○	○	○
Aufgrund der sexuellen Orientierung (z.B. schwul, lesbisch, bisexuell)	○	○	○
Aufgrund eines niedrigen Bildungsstandes	○	○	○
Aufgrund eines geringen Einkommens	○	○	○
Aus einem anderen Grund	○	○	○

Falls Sie "aus einem anderen Grund" angegeben haben, welcher war das?

7 Psychische Gesundheit 1

Psychische Gesundheit

Gesundheitsfragebogen

Wie oft fühlten Sie sich im Verlauf der letzten 2 Wochen durch die folgenden Beschwerden beeinträchtigt?

	Überhaupt nicht	An einzelnen Tagen	An mehr als der Hälfte der Tage	Beinahe jeden Tag
1. Nervosität, Ängstlichkeit oder Anspannung	○	○	○	○
2. Nicht in der Lage sein, Sorgen zu stoppen oder zu kontrollieren	○	○	○	○
3. Übermäßige Sorgen bezüglich verschiedener Angelegenheiten	○	○	○	○
4. Schwierigkeiten zu entspannen	○	○	○	○
5. Rastlosigkeit, so dass Stillsitzen schwer fällt	○	○	○	○
6. Schnelle Verärgerung oder Gereiztheit	○	○	○	○
7. Gefühl der Angst, so als würde etwas Schlimmes passieren	○	○	○	○

Aktuelles Befinden

Wie oft fühlten Sie sich im Verlauf der letzten 2 Wochen durch die folgenden Beschwerden beeinträchtigt?

	Überhaupt nicht	An einzelnen Tagen	An mehr als der Hälfte der Tage	Beinahe jeden Tag
1. Wenig Interesse oder Freude an Ihren Tätigkeiten	○	○	○	○
2. Niedergeschlagenheit, Schwermut oder Hoffnungslosigkeit	○	○	○	○
3. Schwierigkeiten, ein- oder durchzuschlafen, oder vermehrter Schlaf	○	○	○	○
4. Müdigkeit oder Gefühl, keine Energie zu haben	○	○	○	○
5. Verminderter Appetit oder übermäßiges Bedürfnis zu essen	○	○	○	○
6. Schlechte Meinung von sich selbst; Gefühl, ein:e Versager:in zu sein oder die Familie enttäuscht zu haben	○	○	○	○
7. Schwierigkeiten, sich auf etwas zu konzentrieren, z. B. beim Zeitunglesen oder Fernsehen	○	○	○	○
8. Waren Ihre Bewegungen oder Ihre Sprache so verlangsamt, dass es auch anderen auffallen würde? Oder waren Sie im Gegenteil „zappelig“ oder ruhelos und hatten dadurch einen stärkeren Bewegungsdrang als sonst?	○	○	○	○
9. Gedanken, dass Sie lieber tot wären oder sich Leid zufügen möchten	○	○	○	○

7.1.1 Hinweis Suizidalität

Wir haben Ihnen bereits erklärt, dass alle Antworten dieser Befragung anonym gespeichert werden. Sie haben bei einer vorherigen Frage angegeben, dass Sie mindestens an einzelnen Tagen (oder häufiger) Gedanken an den Tod haben oder daran, sich Leid zufügen zu möchten.

Wenn Sie befürchten, sich etwas anzutun oder Gedanken an den Tod haben, sollten Sie ***unbedingt*** *professionelle Hilfe in Anspruch nehmen.*

Wenn Sie sich in einer akuten Krise befinden, wählen Sie bitte die 112 oder begeben Sie sich (z. B. auch in Begleitung einer vertrauten Person) in das nächste Krankenhaus!

In weniger dringenden Fällen können Sie nach Bearbeitung des Fragebogens einen Beratungstermin mit uns vereinbaren oder sich an die Telefonseelsorge unter 0800 111 0 111 oder 0800 111 0 222 oder 116 123 wenden.

Bitte bestätigen Sie, dass Sie den Hinweis zur Kenntnis genommen haben

○ Ich habe den Hinweis gelesen und zur Kenntnis genommen

8 Psychische Gesundheit 2

Fragen zum Alkoholkonsum

	Nie	Etwa 1 mal pro Monat	2-4 mal pro Monat	2-3 mal pro Woche	4 mal oder öfter pro Woche
Wie oft trinken Sie Alkohol?	○	○	○	○	○

Fragen zum Alkoholkonsum

	1 oder 2	3 oder 4	5 oder 6	7 bis 9	10 oder mehr	Ich trinke keinen Alkohol
Wenn Sie an einem Tag Alkohol trinken, wie viele alkoholhaltige Getränke trinken Sie dann typischerweise? (Ein Glas Alkohol entspricht: 0,33 l Bier, 0,25 l Wein oder Sekt, 0,02 l Spirituosen.)	○	○	○	○	○	○

Fragen zum Alkoholkonsum

	Nie	Seltener als 1 mal pro Monat	1 mal pro Monat	1 mal pro Woche	Täglich oder fast täglich
Wie oft haben Sie an einem Tag mehr als 6 alkoholische Getränke getrunken? (Ein Glas Alkohol entspricht: 0,33 l Bier, 0,25 l Wein oder Sekt, 0,02 l Spirituosen.)	○	○	○	○	○

Fragen zum Drogen-/Substanzkonsum

	Nie	Etwa 1 mal pro Monat	2-3 mal pro Monat	2-4 mal pro Woche	4 mal oder öfter pro Woche
Wie oft konsumieren Sie Drogen/ Substanzen?	○	○	○	○	○

Körperbild und Essverhalten

Während der letzten vier Wochen (28 Tage)...

	Überhaupt nicht		Leicht		Mäßig		Deutlich
1. Wie unzufrieden waren Sie mit Ihrem Gewicht?	○	○	○	○	○	○	○
2. Wie unwohl haben Sie sich gefühlt, wenn Sie Ihren Körper gesehen haben (z. B. im Spiegel, Ihr Spiegelbild im Schaufenster, beim Ausziehen, Baden oder Duschen)?	○	○	○	○	○	○	○

Körperbild und Essverhalten

Während der letzten vier Wochen (28 Tage)...

	niemals	in seltenen Fällen	in weniger als der Hälfte der Fälle	in der Hälfte der Fälle	in mehr als der Hälfte der Fälle	in den meisten Fällen	jedes Mal
3. In wie vielen der Situationen, in denen Sie gegessen haben, hatten Sie wegen der Auswirkungen auf Ihre Figur oder Ihr Gewicht Schuldgefühle (d. h. das Gefühl, etwas Falsches getan zu haben)? (Zählen Sie Essanfälle nicht mit.)	○	○	○	○	○	○	○

Körperbild und Essverhalten

Während der letzten vier Wochen (28 Tage)...

	kein Tag	1-5 Tage	6-12 Tage	13-15 Tage	16-22 Tage	23-27 Tage	jeden Tag
4. Haben Sie bewusst versucht, die Nahrungsmenge, die Sie essen, zu begrenzen, um Ihre Figur oder Ihr Gewicht zu beeinflussen (unabhängig davon, ob es Ihnen tatsächlich gelungen ist)?	○	○	○	○	○	○	○
5. Haben Sie versucht, Nahrungsmittel, die Sie mögen, von Ihrer Ernährung auszuschließen, um Ihre Figur oder Ihr Gewicht zu beeinflussen (unabhängig davon, ob es Ihnen tatsächlich gelungen ist)?	○	○	○	○	○	○	○
6. Hat das Nachdenken über Nahrung, Essen oder Kalorien es Ihnen sehr schwer gemacht, sich auf Dinge zu konzentrieren, die Sie interessieren (z. B. arbeiten, einem Gespräch folgen oder lesen)?	○	○	○	○	○	○	○
7. Haben Sie sich dick gefühlt?	○	○	○	○	○	○	○
8. Hatten Sie einen starken Wunsch abzunehmen?	○	○	○	○	○	○	○

9 Nutzung von Hilfsangeboten

Nutzung von Hilfsangeboten

Welche Art von Angeboten würden Sie in Anspruch nehmen, wenn Sie psychische Probleme hätten?

	sehr unwahrscheinlich			sehr wahrscheinlich
Psychiater:in	○	○	○	○
Psychotherapie	○	○	○	○
Heilpraktiker:in für Psychotherapie	○	○	○	○
Psychologische/psycho-soziale Beratung	○	○	○	○
Aufenthalt in einer psychiatrischen/psycho-somatischen Klinik	○	○	○	○
Online Hilfsangebote bei psychischen Problemen (z.B. Apps, Selbstmanagement-Programme)	○	○	○	○
Selbsthilfebücher/-ratgeber	○	○	○	○
Andere, und zwar: ______	○	○	○	○

Welche der folgenden Angebote zur Hilfe bei psychischen Problemen haben Sie schon einmal bzw. im letzten Jahr in Anspruch genommen? *(Mehrfachnennungen möglich)*

	Habe ich schon in Anspruch genommen	Habe ich im letzten Jahr in Anspruch genommen	Nicht zutreffend
Psychiater:in	○	○	○
Psychotherapie	○	○	○
Heilpraktiker:in für Psychotherapie	○	○	○
Psychologische/psycho-soziale Beratung	○	○	○
Aufenthalt in einer psychiatrischen/psycho-somatischen Klinik	○	○	○
Online Hilfsangebote bei psychischen Problemen (z.B. Apps, Selbstmanagement-Programme)	○	○	○
Selbsthilfebücher/-ratgeber	○	○	○
Andere, und zwar ______	○	○	○

Welche Beratungs- und Unterstützungsangebote bietet Ihre Hochschule Ihres Wissens nach an?

	Ja	Nein	Weiß ich nicht
Zentrale Studienberatung	○	○	○
Psychologische/Psychosoziale/Psycho-therapeutische Beratung für Studierende	○	○	○
Beratungsstellen für Studierende mit Kind	○	○	○
Digitale Angebote	○	○	○
Sonstige, und zwar: ______	○	○	○

Welche Beratungs- und Unterstützungsangebote Ihrer Hochschule haben Sie bereits genutzt?

	einmal in Anspruch genommen	mehrfach in Anspruch genommen	Nie
Zentrale Studienberatung	○	○	○
Psychologische/Psychosoziale/Psychotherapeutische Beratung für Studierende	○	○	○
Beratungsstellen für Studierende mit Kind	○	○	○
Digitale Angebote	○	○	○
Sonstige, und zwar: ______	○	○	○

10 Gewissenhafte Bearbeitung

Um die Aussagekraft der Studie sicherzustellen würden wir gerne wissen, ob Sie die Befragung sorgfältig und gewissenhaft ausgefüllt haben.

Ihnen entstehen keine Nachteile, unabhängig davon, welche Antwort Sie auswählen. Die Frage dient lediglich der Sicherstellung der Datenqualität.

- ○ Nein, ich habe den Fragebogen nicht sorgfältig und gewissenhaft ausgefüllt.
- ○ Ja, ich habe den Fragebogen sorgfältig und gewissenhaft ausgefüllt.

11 Angebot_erfahren

Gerne würden wir erfahren, wie Sie von unserem Angebot erfahren haben.

- ○ Rundmail eines E-Mail-Verteilers
- ○ Newsletter einer Organisation, Initative, Verein o.ä.
- ○ Social-Media-Post
- ○ Flyer/Postkarten
- ○ Plakate
- ○ Empfehlung von Freunden oder Familienangehörigen
- ○ Empfehlung durch andere Hilfestellen
- ○ Andere ______

12 Endseite

Vielen Dank für Ihre Teilnahme!

Einige Fragen bezogen sich direkt auf Ihre psychische Gesundheit. Wenn es Ihnen nicht gut geht, Sie Gesprächsbedarf oder ein Anliegen haben, möchten wir Sie dazu einladen, unser Unterstützungsangebot zu nutzen.

Wenn Sie sich jedoch in einer akuten Krise befinden, wählen Sie bitte die 112 oder begeben Sie sich (z. B. auch in Begleitung einer vertrauten Person) in das nächste Krankenhaus!
