# Supplementary material for: Feasibility, acceptance, user satisfaction, and differences between users and non-users of an online-based support service for University students
Source: Discov Ment Health. 2026 Nov 11;6(1):223. doi: 10.1007/s44192-026-00648-5 (PMC13755097; doi:10.1007/s44192-026-00648-5)
Supplement: Supplementary file 3 [file 44192_2026_648_MOESM3_ESM.pdf]

10. Liegt bei Ihnen eine studienerschwerende Beeinträchtigung vor?

X Ja, und zwar (und jeweils anklickbar):

- Psychische Erkrankung (z.B. Depression, Essstörung)
- Körperlich länger andauernde/chronische Erkrankung (z.B. Rheuma, Multiple Sklerose, Darmerkrankung)
- Teilleistungsstörung (z.B. Legasthenie, Dyskalkulie)
- Bewegungsbeeinträchtigung (z.B. Einschränkungen beim Gehen, Stehen, Greifen)
- Sehbeeinträchtigung oder Blindheit (z.B. Einschränkungen der visuellen Wahrnehmungsfähigkeit hinsichtlich der Sehschärfe, des Gesichtsfeldes oder der Farbwahrnehmung)
- Hörbeeinträchtigung oder Gehörlosigkeit (z.B. Minderungen des Hörvermögens, Schwerhörigkeit)
- Gleich schwere Mehrfachbeeinträchtigung (Mehr als eine studienerschwerende Beeinträchtigung, von der keine klar dominiert)
- Andere Beeinträchtigung bzw. Erkrankung (Sprechbeeinträchtigung (z. B. Stottern, Tumorerkrankungen, Autismus-Spektrum-Störung, AD(H)S),Migräne)

X Nein, keine.

11a

Nun geht es um das Thema Diskriminierung. Diskriminierung bedeutet, dass eine Person aus bestimmten Gründen schlechter behandelt wird als andere Menschen, ohne dass es dafür eine sachliche Rechtfertigung gibt. Diskriminierung kann auf sehr unterschiedliche Weise ausgeübt werden, etwa durch Beleidigung, Ausgrenzung, sexuelle Belästigung bis hin zu Gewalt. Es handelt sich aber auch um Diskriminierung, wenn Menschen durch Regeln und Gesetze benachteiligt werden.

Sind Sie persönlich in den letzten 24 Monaten in Deutschland aus den folgenden Gründen diskriminiert worden? (Antwortoptionen und dann jeweils ja/nein/keine Angabe bitte nach jeder Antwortoption)

- Aus rassistischen Gründen, wegen der Zugehörigkeit zu einer ethnischen Gruppe oder der Herkunft aus einem anderen Land
- Aufgrund des Geschlechts
- Aufgrund der Geschlechtsidentität (z.B. transsexuell oder intergeschlechtlich)
- Aufgrund der Religion oder Weltanschauung
- Aufgrund einer Behinderung oder chronischen Krankheit
- Aufgrund zu hohen Alters
- Aufgrund zu niedrigen Alters
- Aufgrund der sexuellen Orientierung (z.B. schwul, lesbisch, bisexuell)
- Aufgrund eines niedrigen Bildungsstandes
- Aufgrund eines geringen Einkommens
- Aus einem anderen Grund

11b

(falls anderer Grund genannt): Aus welchem anderen Grund?

Offenes Format/Textfeld
