# Supplementary material for: Feasibility, acceptance, user satisfaction, and differences between users and non-users of an online-based support service for University students
Source: Discov Ment Health. 2026 Nov 11;6(1):223. doi: 10.1007/s44192-026-00648-5 (PMC13755097; doi:10.1007/s44192-026-00648-5)
Supplement: Supplementary file 4 [file 44192_2026_648_MOESM4_ESM.pdf]

T1a Fragebogen zur Zufriedenheit (ZUF-8)

	Ausgezeichnet	Gut	Weniger gut	Schlecht
1. Wie würden Sie die Qualität des Unterstützungsangebots, welches Sie genutzt haben [PLATZHALTER: Nennung des genutzten Angebots], beurteilen?	☐	☐	☐	☐
	**Eindeutig nicht**	**Eigentlich nicht**	**Im Allgemeinen ja**	**Eindeutig ja**
2. Haben Sie die Art von Unterstützung erhalten, die Sie wollten?	☐	☐	☐	☐
	**Fast allen**	**Den meisten**	**Nur wenigen**	**Gar keinen**
3. In welchem Maße hat das Unterstützungsangebot [PLATZHALTER: Nennung des genutzten Angebots] Ihren Bedürfnissen entsprochen?	☐	☐	☐	☐
	**Eindeutig nicht**	**Ich glaube nicht**	**Ich glaube ja**	**Eindeutig ja**
4. Würden Sie einem Freund/einer Freundin das Unterstützungsangebot [PLATZHALTER: Nennung des genutzten Angebots] empfehlen, wenn er/sie eine ähnliche Hilfe benötigen würde?	☐	☐	☐	☐
	**Ziemlich unzufrieden**	**Leicht unzufrieden**	**Weitgehend zufrieden**	**Sehr zufrieden**
5. Wie zufrieden sind Sie mit dem Ausmaß der Hilfe, welche Sie hier erhalten haben?	☐	☐	☐	☐
	**Ja, sie half eine ganze Menge**	**Ja, sie half etwas**	**Nein, sie half eigentlich nicht**	**Nein, sie hat mir die Dinge schwerer gemacht**
6. Hat die Unterstützung, die Sie hier erhielten, Ihnen dabei geholfen, angemessener mit Ihren Problemen umzugehen?	☐	☐	☐	☐
	**Sehr zufrieden**	**Weitgehend zufrieden**	**Leicht unzufrieden**	**Ziemlich unzufrieden**
7. Wie zufrieden sind Sie mit der Unterstützung, die Sie erhalten haben, im Großen und Ganzen?	☐	☐	☐	☐

	Eindeutig nicht	Ich glaube nicht	Ich glaube ja	Eindeutig ja
8. Würden Sie wieder dieses Unterstützungsangebot [PLATZHALTER: Nennung des genutzten Angebots] wahrnehmen, wenn Sie Hilfe bräuchten?	□	□	□	□

➔ **Hinweis Programmierung: Item 8 ZUF-8 für die Post-Befragung ändern in:** „Würden Sie wieder unsere Unterstützungsangebote wahrnehmen, wenn Sie Hilfe bräuchten?“

T1b Fragen zur Nützlichkeit des Angebots

	Besser	Eher besser	Eher schlechter	Schlechter	
1. Wie fühlen Sie sich jetzt, nach der Teilnahme an [PLATZHALTER: Nennung des genutzten Angebots]?	□	□	□	□	
	**Ja**	**Eher ja**	**Eher nein**	**Nein**	
2. Denken Sie zurück an [PLATZHALTER: Nennung des genutzten Angebots]. Haben wir Ihnen mit Ihrem Anliegen helfen können?	□	□	□	□	
